# Benefits and Mechanisms of Exercise Training for Knee Osteoarthritis

**DOI:** 10.3389/fphys.2021.794062

**Published:** 2021-12-16

**Authors:** Chu-Yang Zeng, Zhen-Rong Zhang, Zhi-Ming Tang, Fu-Zhou Hua

**Affiliations:** ^1^Department of Anesthesiology, The Second Affiliated Hospital of Nanchang University, Nanchang, China; ^2^Department of Rehabilitation Medicine, The Third Hospital of Hebei Medical University, Shijiazhuang, China; ^3^School of Rehabilitation, Capital Medical University, Beijing, China; ^4^Department of Orthopedics, Jiangxi Provincial People’s Hospital Affiliated to Nanchang University, Nanchang, China

**Keywords:** knee osteoarthritis, exercise training, mechanisms, inflammatory, pain, strength training, traditional exercise

## Abstract

Knee osteoarthritis is a chronic degenerative disease. Cartilage and subchondral bone degeneration, as well as synovitis, are the main pathological changes associated with knee osteoarthritis. Mechanical overload, inflammation, metabolic factors, hormonal changes, and aging play a vital role in aggravating the progression of knee osteoarthritis. The main treatments for knee osteoarthritis include pharmacotherapy, physiotherapy, and surgery. However, pharmacotherapy has many side effects, and surgery is only suitable for patients with end-stage knee osteoarthritis. Exercise training, as a complementary and adjunctive physiotherapy, can prevent cartilage degeneration, inhibit inflammation, and prevent loss of the subchondral bone and metaphyseal bone trabeculae. Increasing evidence indicates that exercise training can improve pain, stiffness, joint dysfunction, and muscle weakness in patients with knee osteoarthritis. There are several exercise trainings options for the treatment of knee osteoarthritis, including aerobic exercise, strength training, neuromuscular exercise, balance training, proprioception training, aquatic exercise, and traditional exercise. For Knee osteoarthritis (KOA) experimental animals, those exercise trainings can reduce inflammation, delay cartilage and bone degeneration, change tendon, and muscle structure. In this review, we summarize the main symptoms of knee osteoarthritis, the mechanisms of exercise training, and the therapeutic effects of different exercise training methods on patients with knee osteoarthritis. We hope this review will allow patients in different situations to receive appropriate exercise therapy for knee osteoarthritis, and provide a reference for further research and clinical application of exercise training for knee osteoarthritis.

## Introduction

Knee osteoarthritis (KOA) is a chronic degenerative disease which often causes disability and pain (Madry et al., 2016); especially in people over 50 years of age (Abbassy et al., 2020). Genetic mutations, obesity, trauma, aging local biomechanical factors, and hormones are vital risk factors of KOA (Martel-Pelletier et al., 2016), which can damage in any joint tissue, but mainly cause cartilage destruction, subchondral bone alteration, and synovial inflammation. Moreover, KOA always reduces patients’ ability to perform activities of daily living and work, which causes a severe economic burden on society (Mesa-Castrillon et al., 2021). Therefore, a treatment that can effectively against the degeneration associated with KOA will significantly benefit both patients and society.

Currently, multiple therapy options, including pharmacotherapy, physiotherapy, surgery, and rehabilitation, are available to treat KOA in clinic (Michael et al., 2010). However, pharmacotherapy has many side effects, such as congestive heart failure, hypertension, and renal toxicity (Kan et al., 2019). Physiotherapy has its limitations, which should combine with surgery. Appropriate physiotherapy preoperatively and postoperatively can restore quadriceps strength and improve proprioception of patients with KOA after surgery (Henderson et al., 2018). Surgery is not suitable for patients with early-stage KOA. It is very necessary to find a non-surgical treatment to effectively relieve the symptoms of patients with KOA.

Exercise training aims to improve any part of body functions through the patient’s own strength or the assisted operation of the therapist or with the aid of equipment (Sheikh and Vissing, 2019). A systematic review of randomized trials of therapeutic exercise in patients with KOA indicated that exercise can significantly reduce pain, improve physical function and quality of life (Fransen et al., 2015). Furthermore, exercise training may improve cardiorespiratory function, increase muscle strength, stabilize posture, and ameliorate psychological health (Garber et al., 2011). Thus, exercise training is an effective complementary therapy and plays an important role in the treatment for patients with KOA. However, there are few review articles on exercise training for KOA, and lack the exploration of their mechanisms.

In this review, we summarize the related mechanisms and the therapeutic effects of regular exercise training for the treatment of KOA, describes the main clinical symptoms of KOA, so as to provide a reference for further research and clinical application.

We used “osteoarthritis,” “knee osteoarthritis,” “exercise training,” “mechanism,” “inflammation,” and “rat” as key words. Then we searched PubMed CINAHL, and web of science for methodological papers on articles from July 2001 to July 2021, especially recently 5 years. Having examined 723 full articles, we finally selected 185 articles for this review.

## Mechanisms and Symptoms of KOA

### Mechanism of KOA

According to pathogenic progression, osteoarthritis contains primary osteoarthritis and secondary osteoarthritis. Primary osteoarthritis can be classified into three types according to pathophysiological mechanisms, type I, genetically determined; type II, oestrogen hormone dependent; and type III, age related osteoarthritis (Castaneda et al., 2014). The incidence rate of age related osteoarthritis was the highest.

Not simply articular cartilage damage, KOA is a disease of whole knee joint, involving subchondral bone, capsule, ligaments, synovial membrane, and periarticular muscles (Hunter and Bierma-Zeinstra, 2019). Normal wear and tear, abnormal mechanical loading, injury, and aging are common causes to damage articular cartilage (Xia et al., 2014).

At the early stages of KOA, articular cartilage is still intact. But the molecular composition and organization in the extracellular matrix has altered first (Goldring and Goldring, 2010), which causes a change in water-binding capacity with a reduced mechanical strength (chondromalacia), and leading to a higher deformation of the cartilage under load (Ryd et al., 2015). In the regions of cartilage damage, subchondral plate and subarticular spongiosa thickness progressive increase (Madry et al., 2016), which diminish its biomechanical properties. In addition, Hoffa fat pad will suffer from inflammation, quadriceps femoris become weakness, and knee joint ligament will be laxity (Mahmoudian et al., 2021).

During the progressive stages of KOA, the material properties and structural integrity of the articular surface and underlying hyaline cartilage deteriorate gradually (Goldring and Goldring, 2010). In subchondral bone, the changes include progressive increase in subchondral plate thickness, modification in the architecture of subchondral trabecular bone, and formation of new bone at the joint margins (Goldring and Goldring, 2010). Mechanical and structural changes in meniscal entheses may contribute to meniscal tear, avulsion, and extrusion (Abraham et al., 2014). In infrapatellar fat pad, inflammation enhanced, IL-1β increased, and macrophages raise, which can aggravate cartilage degeneration (Bastiaansen-Jenniskens et al., 2012).

Furthermore, changes in the composition and structure of the articular cartilage further stimulate chondrocytes to produce more catabolic factors involved in cartilage degradation (Xia et al., 2014). The most important catabolic factors are two families member of metalloproteases: the matrix metalloproteinases (MMPs) and the ADAMTSs (a disintegrin and a metalloprotease with thrombospondin motifs). They are key molecules in the extracellular matrix produced by activated chondrocytes of osteoarthritic joints, which are responsible for the degradation of the major components of articular cartilage (Mort and Billington, 2001). As the process continues, increased catabolic activity is related to enhanced production of degradative proteinase genes, which could result in gradual loss of proteoglycans followed by type II collagen degradation (Goldring and Goldring, 2010). Then cartilage integrity is disrupted, and the water content of hyaline cartilage is increased (Adatia et al., 2012). The articular chondrocytes undergo apoptosis and the articular cartilage eventually be completely lost (Loeser et al., 2016). The cartilage serves to decrease friction and distributes the force exerted by loads evenly onto the underlying bone. The total loss of cartilage will reduce joint space, followed by friction between bones (Loeser et al., 2016). At the end stage of KOA, patients appear pain, stiffness, swelling, and limited joint mobility and other symptoms.

A study showed that injection of senescent chondrocyte in a healthy joint is sufficient to promote cartilage damage in an osteoarthritis-like fashion in rat (Xu et al., 2017). Aging decreases the responsiveness of the chondrocytes to growth factors, which influence the catalytic and anabolic metabolism of chondrocytes (Rahmati et al., 2017). Composition of chondrocyte and the extracellular matrix will change with age, which will lead to chondrocyte gradual loss the ability to repair the damage (Rahmati et al., 2017).

Higher mid-stance transverse plane moments could be contributing to higher shear forces in the joint, which can lead to degenerative processes in osteoarthritis cartilage (Astephen Wilson and Kobsar, 2021). Obesity and metabolic syndrome as risk factors, *via* a cumulative influence of the metabolic disorders, accelerate the structural degenerative progress of osteoarthritis (Courties et al., 2019). In addition, in early-stage of KOA, innervation of the articular cartilage and osteophytes is associated with their invasion by blood vessels and nerves (Suri et al., 2007). In addition, estrogen *via* estrogen receptor mediates osteoarthritis cartilage degradation and accelerates the progression of cartilage loss (Tang et al., 2021).

**Figure 1:**
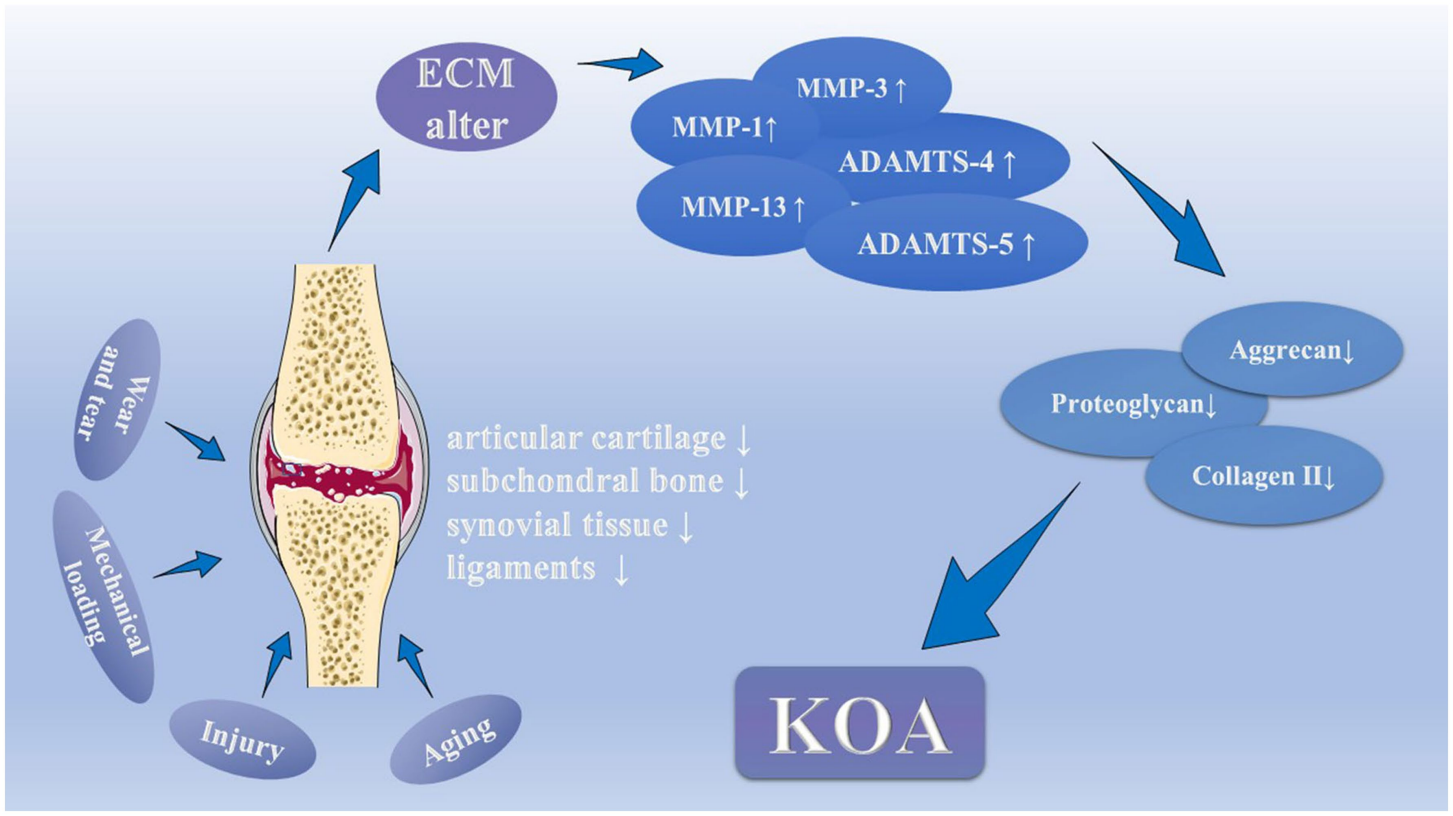
The pathology and pathophysiology of Knee osteoarthritis (KOA). Normal wear and tear, abnormal mechanical loading, injury, and aging are common causes to damage articular cartilage, as well as subchondral bone, synovial tissue and ligaments, which could change the molecular composition and organization in the extracellular matrix. Under the stimulation, injured chondrocytes produce the matrix metalloproteinases (MMP-1, MMP-3, and MMP-13) and the ADAMTSs (ADAMTS-4 and ADAMTS-5). They thus contribute to declining levels of proteoglycans, aggrecan, and type II collagen in the cartilage matrix by inhibiting the synthesis of key components of the extracellular matrix, which eventually leads to cartilage degeneration.

In short, inflammation plays a central role in the development of KOA. Under the stimulation of inflammatory molecules and cytokines, the microenvironment within the joints is gradually destroyed. Which leads to injury of cartilage, subchondral bone, meniscus, and even infrapatellar fat pad, finally patients suffer from KOA (Figure 1). At the same time, mechanobiology, aging, metabolic disorders, hormonal alterations, and vascular and neural invasion also made outstanding contributions to the development of KOA.

### Symptoms of KOA

Patients with KOA usually experience a variety of symptoms, which disturb their daily activities. Four main signs are predominant, including pain, stiffness, reduced joint motion, and muscle weakness (Sharma, 2021).

#### Pain

Pain is a leading feature and one of the most severe disabling symptoms of KOA. The pain of KOA is intermittent and associated with weight-bearing (Hawker et al., 2008; Hunter and Bierma-Zeinstra, 2019). When the affected knee is put in motion, the pain usually becomes worse, but improves when the knee is at rest (Michael et al., 2010). As KOA aggravate, pain will appear at rest and at night, even interfere with sleep (Sharma, 2021). Trouvin et al. (2019) found that in lower limb OA, pain is mostly stable in a long time, and pain is better accepted when stable.

In the synovial fluid of patients with KOA who experience pain, inflammatory molecules, such as interleukin (IL)-6, tumor necrosis factor (TNF)-α, and MMP-13 are constantly elevated (Runhaar et al., 2019). After treatment, the inflammatory molecules significantly reduced (Schell et al., 2017). Therefore, inflammatory molecules in synovial fluid play a vital role in the occurrence of KOA-associated pain.

When tissue damaged during joint degeneration, these nerve fibers distributed in the deep layers of cartilage and subchondral bone will transmit sensation to the brain and crate pain (Adatia et al., 2012). Furthermore, bone attrition considered as flattening or depression of the articular cortex is associated with pain (O’Neill and Felson, 2018). Osteoarthritis patients reporting severe osteoarthritis pain have neuropathic pain-like symptoms, and develop central sensitization. Development of novel compounds targeting molecular pathways implicated in central sensitization may provide improved pain management in advanced osteoarthritis patients (Havelin et al., 2016). In osteoarthritis joint, indirect neuro-immune signaling may occur when innate immune cells produce algogenic factors, such as chemokines and cytokines, which act on the pain pathway (Miller et al., 2020). The transient receptor potential vanilloid (TRPV)-4 ion channel transduces mechanical loading of articular cartilage *via* the generation of intracellular calcium ion transients. Moreover, loss of TRPV4-mediated cartilage mechanotransduction in adulthood could delay the progress of aging-associated osteoarthritis (O’Conor et al., 2016).

Many patients with KOA stop participating in sports and leisure activities because of pain (Gay et al., 2016). Due to the pain, they are afraid to flex and stretch the knee joint, and gradually develop joint mobility disorders, which will diminish their quality of life, and even leads to physical disability (Focht, 2006). Patients with KOA develop neuropathic pain experiencing higher levels of pain, which are significant predictors of risk for all-cause death (Syx et al., 2018). Intense pain may reflect a high degree of joint degeneration (Perrot, 2015), and requiring adequate treatment urgently.

Some studies found that increasing muscle strength and decreasing weight-bearing indexes of the quadriceps could relieve joint pain efficiently (Muraki et al., 2015; Alkhawajah and Alshami, 2019). In addition, KOA patients with pain usually appear long-term depression. Therefore, improving psychological symptoms may be an essential treatment to alleviate pain of KOA (Rathbun et al., 2018).

In summary, pain is intermittent and stable, which is mainly associated with inflammatory stimulation and nerve conduction. The level of inflammatory molecules is related to pain relief. Mechanosensory ion channels, neuro-immune signaling, and central sensitization play a vital important role of neuropathic pain. As the most significant symptoms that disrupted KOA patient’s life, pain relief is their primary demand for seeking treatment for KOA. Muscle strength and psychology improvements could be critical directions for the treatment of KOA-associated pain.

#### Stiffness

Stiffness is a common complaint of patients with KOA (Oatis et al., 2006). It usually appears in the morning on first waking and lasts less than 30 min. Long-term synovitis leads to cell proliferation and increase synthesis of matrix proteins (collagen types I, III, and VI), resulting in mutual adhesion, arthrofibrosis, and gradual joint dysfunction (Mayr and Hochrein, 2015). In the gait weight-bearing phase, changes in knee stiffness during walking are mainly due to decreased knee flexion in patients with KOA, while shifts in external knee flexion account for less than internal knee flexion (Dixon et al., 2010). Gustafson et al. (2016) believe that knee stiffness is a self-compensatory and protective effect when patients with KOA develop joint instability. There is a significant inverse relationship between the symptoms of knee instability and passive mid-range knee stiffness (Creaby et al., 2013), while the relationship between active stiffness and knee joint stability requires further research. Further, Fukutani et al. (2016) found that when the cutoff was K/L grade 2, varus thrust is significantly associated with pain and stiffness.

In summary, KOA-associated stiffness aggravate gradually, and this stiffness is a self-compensatory and protective effect of joint instability.

#### Reduced Joint Motion

Reduced joint motion of the knee is primary and essential manifestation of knee instability symptoms (Chaudhari et al., 2019). Predicting radiographic KOA from range of motion may be possible in the future by further studies of larger sample size conducted in different populations (Ersoz and Ergun, 2003). KOA patients’ knee range of motion is usually limited to different phases (van Dijk et al., 2009). In the early stage of KOA, the range of motion limitation always appears in the end-range motion. As KOA worsens, the joint range of motion becomes smaller (Taylor et al., 2014). Patients with KOA often complain of knee extension and flexion disorders when visiting the doctor (Ersoz and Ergun, 2003). Leon et al. (2005) found that KOA patients with stenosis of the intercondylar notch mixed type have significantly limited joint mobility. van der Esch et al. (2014) found that joint activity limitations are always complimented by muscle weakness; long-term joint activity limitations can lead to muscle weakness and thus exacerbate joint activity limitations. Strengthening the quadriceps can improve joint activity to some extent (Alkhawajah and Alshami, 2019). Furthermore, patients with early-stage KOA have decreased axial tibial rotation excursion, while patients with end-stage KOA have increased knee adduction (Nagano et al., 2012). Campbell et al. (2020) found that knee flexion contractures are associated with worse pain, stiffness, and dysfunction in a severity-dependent manner in patients with KOA.

In summary, reduced joint motion is associated with joint instability, and it is mainly a manifestation of the narrowing of the inner and outer gaps of the knee joint. Patients with KOA often suffer extension and flexion disorders. Pain, stiffness, and muscle weakness are all related to reduce joint mobility. Increasing muscle strength can be a method to alleviate reduced joint mobility.

#### Muscle Weakness

Muscle weakness is a characteristic of patients with KOA (de Zwart et al., 2015), and is a better predictor of disability than pain or joint space narrowing (Roos et al., 2011). Most adults attain their peak muscle strength in their mid-20s and maintain this level until their 60s, but in their 80s, their muscle strength drops to only half of their peak (Latham and Liu, 2010). Muscle weakness may be caused by muscle dysfunction and may be a risk factor for the progression of KOA (Coudeyre et al., 2016). The most apparent muscle weakness is the decrease in extension and flexion strength (Heiden et al., 2009). Extensor weakness is common in patients with KOA, especially quadriceps weakness, which could lead to an increased risk of functional limitation and disability (Latham and Liu, 2010; Jegu et al., 2014). There is an atrophy of the type I and type II fibers of the vastus medialis muscle in patients with end-stage KOA who underwent total knee replacement (Fink et al., 2007). Ikeda et al. (2005) found decreases in the muscle cross-sectional area in patients with early-stage KOA. Decreased muscle strength can increase the risk of falls by decreasing knee stabilizers and proprioceptors (de Zwart et al., 2015). Moreover, knee extensor muscle weakness is related to an increased risk of developing KOA in both men and women (Oiestad et al., 2015; Dell’isola et al., 2018). Long-term weakness of the quadriceps can accelerate the progression of degenerative KOA (Roos et al., 2011). A 6-year cohort study showed that increasing knee muscle strength can prevent the development of KOA-related dysfunction (Latham and Liu, 2010). Increasing the strength of the quadriceps and resisting muscle weakness can relieve the degeneration associated with KOA (Segal et al., 2012).

Muscle weakness is associated with aging and muscle dysfunction, and it can decrease the stability of the knee and accelerate the progression of KOA. Improving muscle weakness, especially in the quadriceps, is significant for the treatment of KOA.

**Figure 2:**
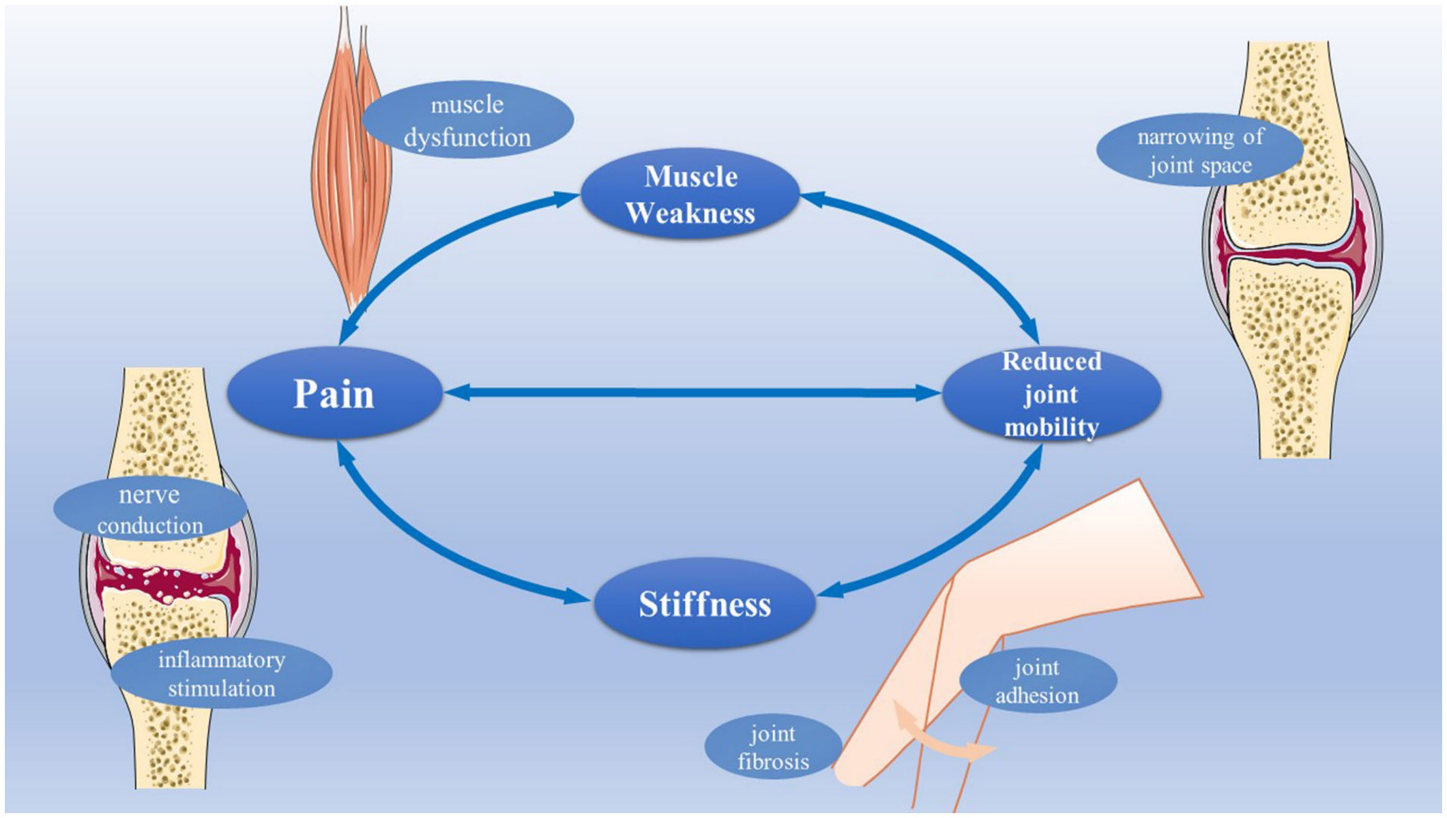
The main symptoms of patients with KOA. The main symptoms of patients with KOA include pain, stiffness, muscle weakness, and reduced joint mobility. Pain is always associated with inflammatory stimulation, and never conduction. Joint fibrosis and joint adhesion are related to stiffness. Muscle dysfunction can cause muscle weakness. Joint space narrowing is related to reduced joint mobility.

Pain, stiffness, joint dysfunction, and muscle weakness are the essential symptoms of KOA, and they are interrelated, not independent (Figure 2). Clinically, our primary treatment goal is to delay the degeneration associated with KOA, reduce the four symptoms, and maximize the function of the knee. Excepting pharmacotherapy and surgery, exercise training may be one of the ways to improve the above symptoms based on their root causes.

## Exercise Training and KOA

### Exercise Training in Animal Experiments

In recent years, a growing number of people have begun to study the mechanisms of exercise training in KOA. Exercise training could effectively increase muscle cross-sectional area and decrease muscle fiber density in experimental animals with KOA (Assis et al., 2015). Increasing the ultimate load supported during the exercise training, the biomechanical characteristics and the structure of the tendon in experimental animals can be improved (Bezerra et al., 2012). Four weeks of regular exercise training can alleviate cartilage degeneration in model rats with KOA (Fallah Mohammadi et al., 2013). There is a biological and biomechanical link between the cartilage and subchondral bone, and that gentle short-term treadmill walking can through inhibiting the increase in osteocyte death to protect the chondrocytes in rat model (Iijima et al., 2015). Four-week treadmill training could alleviate the subchondral bone loss and remodeling, and reprogram the cartilage-subchondral unit (Hao et al., 2021).

Furthermore, aerobic exercise can reduce the expression in IL-1β, caspase-3, and MMP-13, and prevent the degeneration of cartilage caused by KOA in model rats (Assis et al., 2016). Resistance training can decrease MMP-2 activity in quadriceps tendon in a rat model of osteoarthritis (Vasilceac et al., 2021). Treadmills and wheel exercise can decrease the levels of IL-1β, IL-6, and TNF-α, and regulate JNK/NF-ΚB signaling to prevent inflammation in model rats with KOA (Chen et al., 2020). Moderate physical exercise can prevent type B synovial cell dysfunction in rats with early-stage osteoarthritis, and delay the progress of the disease (Castrogiovanni et al., 2019). An animal experiment indicated that, at very early stages of cartilage damage, early intervention by swimming provides better effects than delayed intervention when post-traumatic osteoarthritis already developed (Hsieh and Yang, 2018).

In addition, Allen et al. (2017) found that 4 weeks of treadmill exercise can reverse tactile hypersensitivity and weight asymmetry, and persistent pain in KOA model rats caused by monosodium iodoacetate. Moreover, exercise training has a potential bone stabilizing effect of the osteoarthritis joint (Allen et al., 2017). Cormier et al. (2017) found that voluntary exercise may protect against OA pain, the effect varies as a function of prior exercise duration, and is associated with distinct trabecular bone modifications.

**Figure 3:**
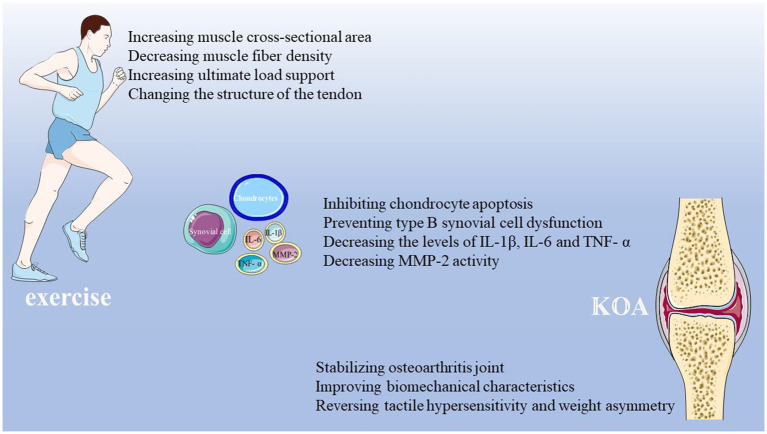
The involved mechanisms for exercise training in treatment of KOA. Existing experimental studies have proved that exercise training has a therapeutic effect on KOA. Exercise training can increase muscle cross-sectional area, decrease muscle fiber density, increase the ultimate load support, change tendon structure, delay musculoskeletal atrophy, stabilize osteoarthritis joint, inhibit inflammation, decrease MMP-2 activity, rescue synovial cell dysfunction, and prevent cartilage degeneration and the loss of subchondral bone of osteoarthritis joint.

In short, various animal experiments of the exercise training to treating KOA have suggested that exercise training can increase muscle cross-sectional area, decrease muscle fiber density, change the tendon structure, delay musculoskeletal atrophy, stabilize the osteoarthritis joint, inhibit inflammation, rescue synovial cell dysfunction, and prevent cartilage degeneration and the loss of subchondral bone (Figure 3). These studies provide an experimental foundation for the application of exercise training in the treatment of KOA, which may be beneficial for patients with this disease.

### Different Types of Exercise Training

**Figure 4:**
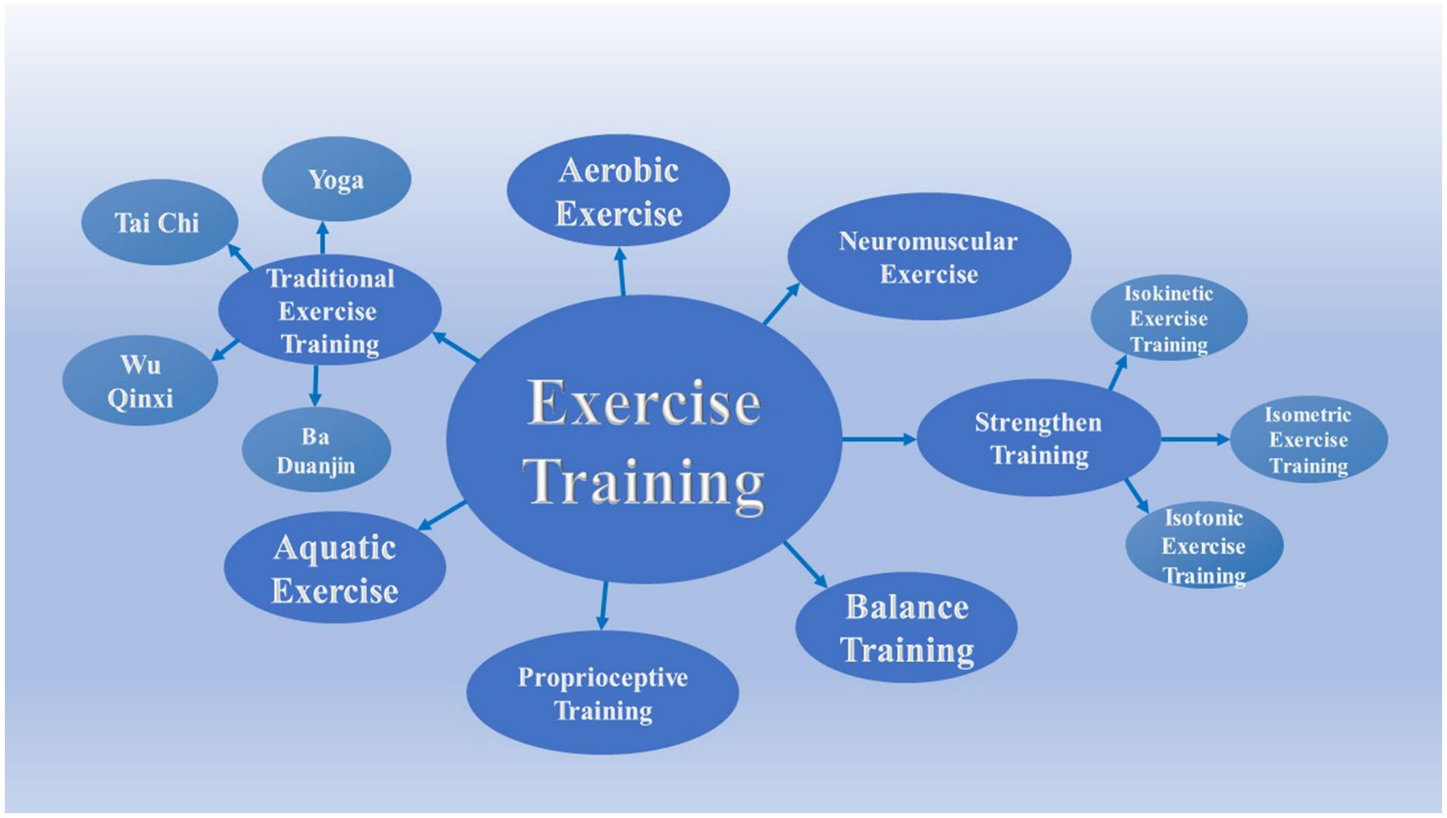
Different exercise training types of KOA. There are several exercise trainings options for treating KOA in the clinic, including aerobic exercise, strength training, neuromuscular exercise, balance training, proprioception training, aquatic exercise, and traditional exercise. Strength training includes isokinetic exercise, isometric exercise, and isotonic exercise. Traditional exercise includes Ba Duanjin, Tai Chi, Wuqinxi, and Yoga.

In clinical, there are several exercise trainings options for the treatment of KOA, including aerobic exercise, strength training, neuromuscular exercise, balance training, proprioception training, aquatic exercise, and traditional exercise (Figure 4). Each kind of exercise training therapy has corresponding therapeutic mechanism and special therapeutic effect on KOA.

#### Aerobic Exercise and KOA

Aerobic exercise is the most convenient exercise training, including walking, jogging, cycling, skating, rhythmic exercises, aerobics, ball games, and rowing (Bartels et al., 2016; Brosseau et al., 2017; Wellsandt and Golightly, 2018). Aerobic exercise has many benefits, such as increasing cardiopulmonary activity, reducing oxidative stress, promoting adipose tissue metabolism, and preventing muscle disuse atrophy in patients with KOA (Ferreira et al., 2015; Gay et al., 2016). Chua et al. (2008) found that aerobic exercise can significantly increase the cartilage oligomeric protein and accelerate the growth of damaged cartilage in patients with KOA. Walking not only can activate T lymphocytes and enhance the body’s immunity in older women with KOA, but also can improve their quality of life and physical performance (Gomes et al., 2016). Moderate supervised aerobic exercise in patients with KOA can improve knee cartilage glycosaminoglycan content, as well as improve pain and function parallel structural (Roos and Dahlberg, 2005). Moreover, Kilic and Tanaka et al. proved that aerobic exercise has a specific therapeutic effect on relieving the pain and dysfunction associated with KOA (Tanaka et al., 2013; Kilic et al., 2020). Retrograde walking can significantly reduce pain, dysfunction, and improve quadriceps strength and performance (Alghadir et al., 2019). Although the results of high-intensity and low-intensity aerobic exercise are consistent, low-intensity aerobic exercise is better for patients with severe KOA (Messier et al., 2021). High-intensity aerobic exercise is effective for patients with mild KOA. In contrast, high-intensity aerobic exercise can lead to more severe damage to cartilage in patients with severe KOA (Multanen et al., 2017). Furthermore, high-intensity interval training is more effective than moderate-intensity training in improving health, body composition, and muscle function in those with chronic disease (Keogh et al., 2017).

Aerobic exercise can promote the metabolism of adipose tissue, prevent muscle atrophy, accelerate the recovery of damaged cartilage, enhance the body’s immunity, and relieve pain. Different intensities of aerobic exercise have different therapeutic effects. Low-intensity aerobic exercise is better for patients with severe KOA, and high-intensity aerobic exercise is more suitable for patients with mild KOA. Furthermore, for mild KOA patients with chronic diseases, high-intensity interval training is better. Doctors should choose the most appropriate treatment for different patients.

#### Strength Training and KOA

Strength training is indispensable for patients with KOA to restore muscle strength. The main functions of strength training are relieving pain, alleviating stiffness, enhancing muscle strength, improving physical function, and increasing the shock absorption ability of the lower extremity muscles during walking (Li et al., 2016; DeVita et al., 2018; Chen et al., 2019; Messier et al., 2021). The types of strength training mainly include isokinetic exercise, isometric exercise, and isotonic exercise (Malas et al., 2013; Sharma, 2021). We will summary the different strength exercise and their effects as follow (Table 1).

**Table 1:** Summary of strength training included in this review.

Study author	Study design	Number of studies/subjects	Intervention studied	Relevant outcome	Main finding
Jegu et al., 2014	RCT	*N* = 80	Eccentric isokinetic strengthening	WOMAC, static postural balance, walking speed, range of knee motion, temporospatial gait parameters, isokinetic tests, and parameters of walking	Increase muscle strength
Ojoawo et al., 2016	RCT	*N* = 45	Proprioceptive and isometric exercises	WOMAC	Reduce pain intensity, enhance physical function, and improve joint stiffness
Malas et al., 2013	RCT	*N* = 61	Strength training	VAS, WOMAC, fascicle length, isokinetic muscle testing, muscle thickness, gait velocity, and function, static balance function	Increase knee extensor strength, increase fascicle length, increase muscle thickness, and influence muscle architecture
Akyol et al., 2010	RCT	*N* = 40	Isokinetic exercise, short-wave	VAS, WOMAC, 6-MWT, isokinetic muscle testing, SF-36, and beck depression index	Relieve pain, reduce disability, increase walking distance, increase muscle strength, increase quality of life, and reduce depression
Samut et al., 2015	Prospective	*N* = 42	Isokinetic and aerobic exercise	VAS, WOMAC, ROM, 6-MWT, functional activity status, isokinetic testing, serum biomarker, and 30 s sit to stand test	Reduce pain, improve physical function, increase muscle strength, and decrease TNF-α, IL-6, and CRP
Cetin et al., 2008	RCT	*N* = 100	Hot pack, short-wave diathermy, TENS, Ultrasound, and Isokinetic muscle-strengthening exercise	VAS, ISK, Ambulation time, and Isokinetic test	Reduce pain, improve walking ability, increase walking speed and function, increase muscle strength, and improve knee extension and flexion
Miyaguchi et al., 2003	Prospective	*N* = 17	Isometric quadriceps exercise	VAS, circumference of the thigh, maximum isometric quadriceps and hamstring forces at 30 and 60° knee flexion, and joint fluid biomarker	Relieve pain, increase muscle strength, increase molecular weight of hyaluronan, increase viscosity of joint fluid, and decrease chondroitin 4-, 6-sulfate concentration in joint fluid
Tok et al., 2011	RCT	*N* = 40	Electrical stimulation, continuous passive motion vs. isometric exercise	VAS, WOMAC, SF-36, knee and thigh circle measurements, isokinetic tests, and dynamic and static balance tests	Increase dynamic and static balance, increase muscle strength, improve pain, and improve quality of life
Anwer et al., 2013	Preliminary	*N* = 43	Isometric exercise, electromyographic biofeedback	Isometric strength of quadriceps	Increase muscle strength
Onigbinde et al., 2017	Prospective	*N* = 21	Isometric quadriceps strengthening training	Quadriceps strength	Increase quadriceps strength
Huang et al., 2003	Prospective	*N* = 135	Isokinetic, isotonic, and isometric muscle-strengthening exercise	VAS, muscle power of leg flexion and extension, and ambulation speed	Relieve pain, decrease disability, increase muscle strength, improve joint stability, improve walking endurance, and increase walking speed
Burrows et al., 2014	RCT	*N* = 33	Acute resistance exercise	Pressure pain threshold, pressure pain tolerance	Increase pain sensitivity, increase pressure pain thresholds
Chang et al., 2012	Prospective	*N* = 41	Elastic-band exercise	VAS, WOMAC, 30s CST, and walking function (10 m walk test, TUG test, and going up-and-down 13-stair test)	Improve lower-extremity function
León-Ballesteros et al., 2020	RCT	*N* = 32	Kinesiotape and quadriceps strengthening with elastic band	WOMAC (pain, stiffness and functionality), VAS	Relief pain, improve functionality, and decrease stiffness
Ferraz et al., 2018	Prospective	*N* = 48	Resistance training with blood flow restriction	WOMAC (Pain, stiffness, and physical function), SF-36, 1-RM test, TST, TUG tests, and Quadriceps cross-sectional area	Improve pain, induce less joint stress, increase muscle strength, increase quadriceps muscle mass, and improve functionality
Segal et al., 2014	RCT	*N* = 45	Blood flow restricted low-load resistance training	Isotonic knee extensor strength, stair climb muscle power, quadriceps volume, and KOOS	Increase muscle strength and volume, increase knee extensor and leg press strength

BMI, body mass index; CRP, C-reactive protein; IL-6, Interleukin-6 ISK; the index of severity for knee osteoarthritis; KOOS, Knee Injury and Osteoarthritis Outcome Score; RCT, randomized controlled trial; SF-36, Medical Outcomes Study Short-Form Health Survey; TNF-α,Tumor Necrosis Factor-α; TST, timed-stands test; TUG test, Timed Up and Go test; VAS, Visual Analog Scale; WOMAC, Western Ontario and McMaster Universities Osteoarthritis Index; 1-RM test, one repetition maximum test; 6-MWT, 6-min walk test; and 30s CST, 30 s Chair Stand Test.

##### Isokinetic Exercise and KOA

Isokinetic exercise refers to exercise training in which muscle strength changes but movement speed does not change (Coudeyre et al., 2016). Isokinetic muscle strengthening is an effective way to promote dynamic muscle strengthening for KOA rehabilitation and has a significant effect on disability and pain (Coudeyre et al., 2016). Samut et al. (2015) found that 6 weeks of isokinetic exercise in patients with KOA can decrease TNF-α, IL-6, and C-reactive protein in patients’ serum, as well as relieve pain, increase functional capacity, and improve muscle strength. A randomized controlled clinical study showed that isokinetic exercise can enhance muscle strength, increase walking distance, and improve quality of life in patients with KOA (Akyol et al., 2010). Moreover, a randomized trial proved that isokinetic eccentric exercise is better than isokinetic concentric exercise for patients with KOA in improving gait, enhancing static equilibrium, and relieving pain (Jegu et al., 2014). By combining isokinetic exercise with a variety of treatment methods, Cetin et al. (2008) tested 100 patients with bilateral KOA and found that KOA patients in the isokinetic exercise group alone experienced the most significant pain alleviation, with a maximum in walking speed and function at 60 and 180 degrees per second of speed, as well as increased muscle strength.

In summary, isokinetic exercise can decrease IL-6 and TNF-α levels in patients’ serum, inhibit inflammation, relieve pain, and increase muscle strength. Further, for the patients with bilateral KOA, the therapeutic effect of isokinetic eccentric exercise is better than that of isokinetic centripetal exercise.

##### Isometric Exercise and KOA

Isometric exercise (also known as static exercise) involves the isometric contraction of the muscle. During muscle contraction, muscle tension increases significantly, while muscle length does not change (Huang et al., 2018). Miyaguchi et al. (2003) found that 12 weeks of exercise of the quadriceps of patients with KOA, through increasing the molecular weight of the hyaluronan and the viscosity of the joint fluid in the knee joint to improve the symptoms of KOA (Miyaguchi et al., 2003). Isometric resistance exercise of the quadriceps of patients with KOA can significantly increase the sensitivity and coordination of proprioceptors in the quadriceps (Topp et al., 2002). Tok et al. (2011) demonstrated that isometric exercise of the quadriceps can improve the dynamic and static balance of patients with KOA. Furthermore, maximum or secondary isometric exercise is benefit in restoring neuromuscular function in patients with KOA (Mau-Moeller et al., 2017). Ojoawo et al. (2016) found that isometric exercise can effectively improve the joint stiffness and physical difficulties associated with KOA. Anwer et al. (2013) demonstrated that isometric exercise can significantly increase muscle strength in both female and male patients. When only the ipsilateral homologous muscle is strengthened, there is a cross-training effect on the contralateral quadriceps in patients with KOA (Onigbinde et al., 2017).

In summary, isometric exercise can increase the hyaluronic acid levels and viscosity of the joint fluid in the joint capsule in patients with KOA. Moreover, isometric exercise has an excellent therapeutic effect on proprioception and muscle strength recovery in patients with KOA. There is no difference in the training effect between different sexes.

##### Isotonic Exercise and KOA

Isotonic exercise (also known as dynamic contraction) refers to exercise training with isotonic contraction. During muscle contraction, muscle tension remains unchanged, but the length of the muscle fibers is shortened or prolonged, resulting in visible movement of the joints. The change in muscle fiber length during muscle contraction can be categorized based on isotonic centripetal exercise (such as jumping) or isotonic centrifugal exercise (such as squatting and walking down stairs). Compared with isometric exercise and isokinetic exercise, isotonic exercise has the most significant effects to relieve pain for patients with KOA (Huang et al., 2003). Furthermore, a clinical trial of 61 patients with KOA showed that isotonic exercise can alleviate pain, stiffness, and improve knee joint function effectively, but it cannot increase quadriceps strength significantly (Malas et al., 2013). In addition, Tanaka et al. (2018) found that the muscle strength of patients with KOA could be effectively improved through low-load isotonic resistance exercise. Moreover, isotonic-centripetal exercise and isotonic-eccentric exercise has the same effect on increasing knee extension and knee flexion muscle strength, as well as relieving pain in patients with KOA (Vincent et al., 2019).

In summary, isotonic exercise is prevalent in people’s daily life. Isotonic exercise can alleviate pain; enhance muscle strength significantly in patients with KOA. The therapeutic effects of different isotonic exercises are almost similar. People could choose different training modes based on their preferences, goals, physical tolerance levels, and equipment availability.

##### Other Strength Exercise and KOA

Apart from above strength exercise training options, many other strength exercise training options are available in hospital. Resistance training is one of the most common rehabilitation training options for patients with KOA, and it is often combined with aerobic exercise, strength training, or aquatic exercise (Kristensen and Franklyn-Miller, 2012; Coudeyre et al., 2016; Munukka et al., 2020). Following resistance exercise, the pain thresholds of patients with KOA changed, and pain sensitivity tolerance decreased (Burrows et al., 2014). There is a significant improvement in function, strength, and mobility after 8 weeks of resistance exercise in patients with KOA (Pazit et al., 2018). Elastic-band exercise is flexible and convenient, an 8 weeks of leg press exercise using elastic bands has been shown to significantly improve lower-extremity function in females with KOA (Chang et al., 2012). However, the existing research does not prove that elastic-band training of the quadriceps femoris results in better pain relief than quadriceps strengthening exercise in patients with KOA (León-Ballesteros et al., 2020). Recently, resistance training with blood flow restriction has attracted the attention of physical therapists and has been applied in patients with KOA. Using a pressure cuff with continuous compression of the proximal portion of the extremity, the cuff occludes venous return from the muscle, and maintains partial arterial flow in the muscle (Cuyul-Vasquez et al., 2020). Resistance training with blood flow restriction can induce less joint stress, relieve pain, increase muscle strength, increase quadriceps muscle mass, and increase functionality in patients with KOA (Ferraz et al., 2018). Moreover, blood flow-restricted low-load resistance training is effective in increasing knee extensor strength and leg press ability in women at risk for KOA (Segal et al., 2014).

In summary, strength training is an essential part of exercise training for patients with KOA. Different strength training options have different effects, but the common feature is increasing muscle strength. Different strength training options may be combined to treat patients with KOA of different stages. This may be an important development direction for the future treatment of KOA.

#### Neuromuscular Exercise and KOA

Neuromuscular exercise can improve balance, muscle activation, functional alignment, and joint stability. The primary purpose of neuromuscular exercise is to achieve compensatory functional stability and improve sensorimotor control (Ageberg et al., 2013). A randomized, single-blind, controlled trial found that neuromuscular exercise can significantly improve cartilage matrix quality and reduce knee-joint loads in patients with mild KOA (Holsgaard-Larsen et al., 2017). Individualized and gradual neuromuscular exercise improves patient-reported outcomes and physical function (such as the ability to move independently and knee extensor strength), even in older patients with severe primary KOA (Ageberg et al., 2013). A randomized controlled trial found that 8 weeks of supervised neuromuscular exercise before total knee replacement can effectively improve the quality of life of KOA patients after operation (Fernandes et al., 2017). Neuromuscular exercise may be the best choice of exercise training for pain relief in KOA patients with inverted thrust (Ageberg and Roos, 2015). Increased medial knee neuromuscular activity is prevalent for exhibiting medial knee joint laxity and varus alignment patients. Increasing neuromuscular exercise can increase the co-contraction, amplitude, and duration of the lateral knee muscles in patients with KOA (Mills et al., 2013). Moreover, neuromuscular exercise has a better therapeutic effect on knee joint loads, pain, and physical function in patients with intra-KOA and varus malalignment than quadriceps strengthening exercise (Bennell et al., 2011). Holsgaard-Larsen et al. (2018) showed that comparing to pharmacotherapy, neuromuscular exercise might be a better choice to relieve long-term symptoms such as swelling and stiffness, dealing with mechanical problems, and avoiding the potential side effects of analgesics and anti-inflammatory drugs. Clausen et al. (2017) first reported that neuromuscular exercise is therapeutic for patients with KOA in the early and mid-stage, but it cannot improve patients’ ability to jump.

Clinically, neuromuscular exercise has a good therapeutic effect on patients with KOA with inverted thrust or varus malalignment. For end-stage KOA patients who require knee arthroplasty, neuromuscular exercise before surgery can effectively relieve postoperative pain. Currently, neuromuscular exercise focuses on patient’s post-knee joint replacement, and more research on neuromuscular exercise for early KOA is needed.

#### Balance Training and KOA

Balance training challenges people to regain their center of gravity during destabilizing movements and to reduce the size of their support base, which requires feedforward and feedback postural control instances and gait tasks (Schlenstedt et al., 2015). There are many forms of balance training, including static balance training, dynamic balance training, balance instrument training, and Virtual Reality (VR) training (Diracoglu et al., 2005; Duque et al., 2013; Takacs et al., 2017). Balance training is necessary for KOA patients with a higher risk of falling; it can reduce the risk of falls in patients with KOA (Levinger et al., 2017; Anderson et al., 2019). The clinical trial showed that preoperative balance training can improve the early postoperative balance but not the perceived functionality of patients with KOA (Blasco et al., 2020). Balance training can improve walking ability and balance performance, alleviate pain, as well as enhance physical function (Doma et al., 2018). Furthermore, progressive and dynamic balance training has a better effect than conventional physiotherapy on improving physical function, range of motion, and balance for patients with KOA (Lee et al., 2021). Biodex balance training is better than traditional exercise programs to improve functional performance, stability, and body sensation, and to reduce swaying and pain in patients with KOA (Javed et al., 2021). A randomized controlled trial proved that dynamic balance training based on visual feedback can alleviate knee pain and joint stiffness, by preventing asymmetric joint alignment, improving the motion of the knee joint, and reducing mechanical friction of the knee (Lee et al., 2020).

In short, balance training is better than traditional physical training to improve the physical function of patients with KOA. Enhancing balance ability, stabilizing motor function, and reducing fall risk are a characteristic effect of balance training.

#### Proprioceptive Training and KOA

In patients with KOA, weakening and damage of the knee muscles, tendons, ligaments, and articular capsule is related to body’s proprioception decreases (Relph and Herrington, 2015). The weakening and damage of proprioceptors make patients’ pain and perception abnormal could lead to the severe consequence of KOA (Bennell et al., 2003). Weak patients with poor proprioceptors have limited joint functional ability (van der Esch et al., 2007). Proprioceptive training takes more significant improvement in proprioception recovery, walking time, and knee extension strength in patients with KOA (Lin et al., 2009). At the same time, proprioceptive training can delay the progression of KOA, such as reducing pain, improving joint and muscle health, and improving the functional quality of patients with early-stage KOA (John Prabhakar et al., 2020). Furthermore, enhancing patients’ walking ability can effectively reduce the basic risk of falls in patients with end-stage KOA (Aljehani et al., 2021). Duman et al. (2012) proprioceptive training has a great effect on improving the accuracy of static balance and proprioception.

In summary, proprioceptive training through activation of proprioceptors improves the condition of KOA. For end-stage KOA, increasing exercise precisely for proprioception and balance dysfunction is necessary. The effect is more pronounced in patients who experience pain when bearing weight.

#### Aquatic Exercise and KOA

Aquatic exercise *via* temperature stimulation and buoyancy of water improve patients’ motor dysfunction (Hinman et al., 2007). Especially weight-bearing loss caused by the buoyancy of water, which play a therapeutic role for patients (Lu et al., 2015). There is a faster effect to decrease knee stiffness in a short period therapy with aquatic exercise than routine rehabilitation training (Munukka et al., 2020). Aquatic exercise with progressive resistance can increase the thickness of the posterior region of interest of the medial femoral cartilage, and improve cardiopulmonary function (Munukka et al., 2016). Further studies revealed that regular swimming can reduce joint pain and stiffness, improve muscle strength and function in middle-aged and older adults with KOA (Alkatan et al., 2016). Compared to land-based exercise, aquatic exercise has fewer side effects and better therapeutic effects (Lund et al., 2008; Lu et al., 2015). Aquatic exercise has a better therapeutic effect for obese postmenopausal women with KOA, not only can alleviate pain, dysfunction, and improves quality of life, but also decrease fat mass (Lim et al., 2010; Yazigi et al., 2013; Waller et al., 2017; Rewald et al., 2020). A randomized controlled trial showed that dance-based aquatic exercise can significantly improve physical function and cardiorespiratory capacity, as well as decrease postexercise heart rate and fatigue in obese postmenopausal women with KOA (Casilda-Lopez et al., 2017). Roper et al. (2013) found that acute aquatic treadmill exercise can be a conservative treatment to improve joint angular velocity and arthritis-related joint pain. Aquatic exercise can significantly improve knee flexibility, strength, and aerobic fitness, and do not worsen the joint condition associated with KOA (Wang et al., 2007). Kunduracilar et al. (2018) found that adding upper extremity and trunk exercises to lower extremity exercises during aquatic exercise training is effective for improving physical function, balance, and pain.

In summary, water temperature stimulation and buoyancy are the advantages of aquatic exercise. Aquatic exercise has a recognized effect on physical function and quality of life of patients with KOA. Moreover, due to the weight-bearing reduction effect of water, aquatic exercise is a good treatment option for KOA patients with a high body mass index.

#### Traditional Exercise and KOA

##### Ba Duanjin and KOA

Ba Duanjin, a traditional Chinese exercise, is described as a mind–body practice that integrates spirit and meditation with slow and gentle postures, as well as musculoskeletal stretching and deep breathing (Zeng et al., 2020). It can alleviate morning stiffness, spinal pain, and fatigue, and it can relieve musculoskeletal pain in elderly people in particular (Li et al., 2019; Xie et al., 2019). An et al. (2008) found that Ba Duanjin is a safe and feasible treatment option for elderly with KOA, as it offers reductions in pain, stiffness, and disability, which can improve patients’ quadriceps strength and aerobic ability. Long-term Ba Duanjin may be viable and safe exercise training for KOA patients to relieve pain and stiffness, and improve physical function (An et al., 2013). A study showed that Ba Duanjin can increase the resting-state functional connectivity of the right lingual gyrus and the right cerebellum/occipital fusiform gyrus/thalamus, as well as decrease the resting-state functional connectivity of the right medial orbital prefrontal cortex, left superior parietal lobule, and left superior temporal gyrus in patients with KOA (Liu et al., 2019a). Liu et al. (2019b) found that patients with KOA have higher dorsolateral prefrontal cortex resting-state functional connectivity on the left side, as well as increase dorsolateral prefrontal cortex resting-state functional connectivity in the left-supplementary motor area and left-temporoparietal junction after participating in Ba Duanjin.

In summary, Ba Duanjin regulates both the downstream opioid energy pathway and the dorsolateral prefrontal cortex (cognitive control) pathway, altering inflammatory blood markers through resting-state functional connectivity and the dorsolateral prefrontal cortex-supplementary motor area resting-state functional connectivity of the reward/incentive system. Furthermore, it has a particular curative effect on patients with KOA, which is associated with decreased joint mobility, and is thus worthy of continued clinical research.

##### Tai Chi and KOA

Tai Chi, a gentle aerobic exercise, is derived from ancient Chinese martial arts that can relax the body and mind (Chen et al., 2016). Compared with other conventional physical therapy, Tai Chi has a better treatment effect on reducing depression (Li et al., 2020). The shortest adequate treatment time of Tai Chi for patients with KOA is 2–5 weeks (Lee et al., 2018). A clinical experiment showed that Tai Chi can change KOA patients’ gait and plantar pressure load pattern during walking (Zhang et al., 2020). You et al. (2021) found that Tai Chi can be an excellent physical training strategy for improving postural control and walking function in older individuals with KOA. Furthermore, Tai Chi has positive effects on muscular activities and proprioception of the leg and ankle, and it can improve balance on both rigid and foam surfaces in older patients with KOA (Ghandali et al., 2017; Hu et al., 2020). Tsai et al. (2013) found that Tai Chi might have a therapeutic effect on elderly people with cognitive impairment and KOA. Hu and Brismee et al. found that Tai Chi can significantly reduce pain and dysfunction, improve KOA patients’ physical and mental health, which can be an alternative to non-drug therapies in rehabilitation programs (Brismee et al., 2007; Hu et al., 2021).

In summary, Tai Chi is a popular mind-body exercise, which can relieve pain, reduce dysfunction of KOA, and it has significant effects on improving depression, exercise gait, and postural stability. However, the effects of Tai Chi are slowly, it always takes more than 2 weeks to get an effect.

##### Wuqinxi and KOA

Wuqinxi is a traditional Chinese exercise that was designed by Hua Tuo at the end of the Eastern Han Dynasty (Guo et al., 2018). It can release muscle tone and increase blood flow, thereby relieving pain. Long-term Wuqinxi can significantly enhance the physical function of chronically ill patients, improving their strength, bone density, balance, joint flexibility, mental vitality, and psychological confidence (Wei et al., 2015). A randomized controlled trial showed that from pretest to follow-up, KOA patients in the Wuqinxi group showed significantly improved, isokinetic knee flexion, and extension strength, timed up and go test, 6-min walk test, 30-s chair stand test, and their pain was much relieved (Xiao et al., 2021). Kang et al. showed that Wuqinxi promotes balance and pain relief in KOA patients more effectively than traditional physiotherapy exercises (Xiao et al., 2020). In addition, Xiao et al. found that the stability test, the static postural stability test, and the dynamic fall index test results of elderly, female KOA patients improved after 24 weeks of Wuqinxi (Xiao and Li, 2021).

In short, Wuqinxi is a very suitable exercise for elderly people, which can enhance the balance of KOA patients, reduce pain, and increase muscle strength. However, there is not enough research on Wuqinxi. More profound and relevant studies are needed.

##### Yoga and KOA

Yoga, a traditional Indian exercise, has apparent effects on psychological and physical health (Field, 2016). Yoga can stimulate baroreceptors, increase vagus nerve activity and serotonin levels, slow cortisol and substance P production, and relieve pain (Melzack and Wall, 1965). A randomized controlled study showed that after 8 weeks of yoga training, the pain, stiffness, and sleep disturbance of elderly female KOA patients were significantly reduced at 20 weeks (Cheung et al., 2014). A randomized controlled trial by Moonaz et al. (2015) found that yoga can safely increase physical activity, improve physical and mental health, and improve health-related quality of life in sedentary patients with arthritis. Lomgpre et al. showed that the yoga postures of squatting and lunging can improve quadriceps strength and minimize exposure to high adduction torque of the knee joint in patients with KOA (Longpre et al., 2015). Compared with traditional exercise-based program, biomechanically-based yoga is better for female patient with KOA to reduce pain, improve physical function and mobility (Kuntz et al., 2018).

In summary, the therapeutic effect of yoga on KOA is noticeable. Breaking down the various postures of yoga and designing more appropriate exercise training programs based on effective yoga postures may be a new direction for the treatment of KOA in the future.

All in all, traditional exercise has been proven to have a certain therapeutic effect on KOA, such as reducing pain, improving joint mobility, improving quality of life, and enhancing physical function (Table 2). However, the evidence and mechanism are not sufficient; we hope there will be more and more studies on traditional exercise.

**Table 2:** Summary of traditional exercise included in this review.

Study author	Study design	Number of studies/subjects	Intervention studied	Relevant outcome	Main finding
An et al., 2008	Prospective	*N* = 28	Baduanjin	WOMAC, SF-36, 6-MWT, Isokinetic Strength of the Knee Extensors (ISKE) and BMI	Relieve pain, reduce stiffness, improve general and emotion health, decrease disability, enhance knee extensors and flexors strength, improve aerobic ability, and lose weight
An et al., 2013	Prospective	*N* = 28	Baduanjin	WOMAC, SF-36, 6-MWT, Isokinetic Strength of the Knee Extensors and Flexors (ISKEF), and BMI	Relieve pain, reduce stiffness, improve general and emotion health, decrease disability, enhance knee extensors and flexors strength, improve aerobic ability, and lose weight
Chen et al., 2019; Liu et al., 2019a	Multiple mode MRI study	*N* = 140	Tai Chi, Baduanjin, stationary cycling, health education	KOOS, functional and structural MRI, and serum biomarkers	Reduce pain, decrease BDNF, IFN-γ, PD-1, and TIM-3, and modulate brain areas known to be involved in the opioidergic and dopaminergic neurotransmitter systems
Liu et al., 2019b	Multiple mode MRI study	*N* = 140	Tai Chi, Baduanjin, stationary cycling, and health education	KOOS, functional MRI, serum biomarkers	Relieve pain, decrease BDNF, INF-γ, PD-1, and TIM-3, and decreased the rsFC between the bilateral DLPFC and bilateral
Lee et al., 2018	Prospective	*N* = 182	Tai Chi, physical therapy exercise	WOMAC, VAS, SF-36, Kellgren and Lawrence grade	Relieve pain, improve physical function
Zhang et al., 2020	Prospective	N = 46	Tai Chi	Plantar load assessment (peak pressure and maximum force)	Increase plantar loads in forefoot
You et al., 2021	Systematic review and meta-analysis	11 studies	Tai Chi	6-MWT, TUG test, and WOMAC	Improve dynamic stability and walking capacity
Ghandali et al., 2017	Prospective	*N* = 20	Tai Chi	Area and mean velocity of CoP, Postural stability and control	Improve motor control and postural stability
Brismee et al., 2007	RCT	*N* = 41	Group and home-based tai chi	VAS, WOMAC, active range of motion for flexion and extension	Reduce pain, improve physical function
Hu et al., 2020	Prospective	*N* = 52	Tai Chi	VAS, WOMAC, and knee and ankle proprioception	Reduce pain, improve ankle and knee proprioception
Tsai et al., 2013	Pilot cluster-randomized trial	*N* = 55	Tai Chi	WOMAC, Get Up and Go test, Sit-to-Stand test, and Mini-Mental State Examination	Reduce pain, improve stiffness, improve physical function, and improve cognitive function
Hu et al., 2021	Systematic review and meta-analysis	16 studies (*N* = 986)	Tai Chi	WOMAC, 6-MWT, dynamic balance, and physiological and psychological health	Reduce pain, maintain mobility, enhance muscle strength, enhance range of joint motion, and ameliorate physical and mental health
Xiao et al., 2021	RCT	*N* = 68	Wuqinxi	WOMAC, Berg Balance Scale, TUG Test, 6-MWT, 30sCST, and isokinetic muscle strength testing of knee flexion and extension	Decline pain, increase knee extensor strength and Knee flexor strength
Xiao et al., 2020	RCT	*N* = 98	Wuqinxi	Berg Balance Scale, TUG Test, 6-MWT, 30sCST, WOMAC, knee extension strength, and knee flexion strength	Decline pain, increase knee extensor strength, and Knee flexor strength
Xiao and Li, 2021	Prospective	*N* = 284	Wuqinxi	Limits of stability tests, static posture stability tests, dynamic fall index tests, WOMAC, and SF-36	Reduce pain, improve balance function, and improve subjective quality of life
Cheung et al., 2014	RCT	*N* = 36	Yoga	WOMAC, QoS, QoL, repeated chair stands, balance, and timed 8 foot walk	Reduce pain, decrease stiffness, improve sleep, and improve physical function
Moonaz et al., 2015	RCT	*N* = 75	Yoga	SF-36, HRQoL	Increase physical activity, improve physical and mental health, and improve quality of life
Longpre et al., 2015	Prospective	*N* = 30	Yoga-based knee strengthening exercises	Muscle Activation, Knee Adduction Moment	Improve leg strength
Kuntz et al., 2018	RCT	*N* = 31	Biomechanically-based yoga	KOOS, ICOAP, self-reported physical function, 6-MWT,30sCST, 40 m W, TUG test, stair ascent test, muscle strength, CESD, and HRQoL	Reduce pain, improve physical function, improve quality of life, increase muscle strength, and improve mobility

BDNF, Brain-derived neurotrophic factor; BMI, body mass index; CoP, Center of Pressure; DLPFC, dorsolateral prefrontal cortex; HRQoL: health-related quality of life; INF-γ, interferon-γ; KOOS, Knee Injury and Osteoarthritis Outcome Score; MRI, magnetic resonance imaging; PD-1, programmed cell death protein 1; QoL, quality of life; QoS, quality of sleep; RCT, randomized controlled trial; SF-36, Medical Outcomes Study Short-Form Health Survey; TIM-3, T-cell Ig-and mucin-domain–containing molecule–3; TUG test, Timed Up and Go test; VAS, Visual Analog Scale; WOMAC, Western Ontario and McMaster Universities Osteoarthritis Index; 6-MWT, 6-min walk test; 30sCST, 30 s Chair Stand Test; 40 m W, 40-meter walk; and CESD, Center for Epidemiological Studies Depression Scale.

## Conclusion

Various exercise training options have been reported to have therapeutic effects on KOA. Among the different exercise interventions, aerobic exercise, which alleviates pain and improves physical function, is the most widely used. Strength training is the most effective exercise therapy against muscle weakness. Neuromuscular exercise and balance training are the best exercise training options to improve proprioception, sensorimotor control, and functional stability. Aquatic exercise has fewer side effects than other exercise training, more and more people are trying aquatic exercise. In addition, many traditional exercises, such as Ba Duanjin, Tai Chi, Wuqinxi, and Yoga are gradually being used to treat KOA. Their effects on the psychology of patients with KOA are noticeable. On the premise of ensuring patient safety, we should provide more individualized exercise prescriptions for patients with different stages of KOA.

However, the current research on exercise training for KOA is not satisfactory. We need more extensive and more profound clinical studies (involving education, traditional exercise, etc.) to confirm the effectiveness of exercise therapy in treating patients with KOA. In addition, combining various exercise therapies to make an optimal treatment plan for different patients would be an important development direction for the treatment of KOA in the future. At present, the mechanisms of exercise training for KOA treatment are poorly understood. We urgently need more animal experiments to prove the principle of efficient treatment, and to promote the development of exercise therapy for KOA treatment in humans.

## Data Availability Statement

The original contributions presented in the study are included in the article/supplementary material; further inquiries can be directed to the corresponding authors.

## Author Contributions

All authors listed have made a substantial, direct and intellectual contribution to the work, and approved it for publication.

## Funding

This work was supported by the Traditional Chinese Medicine of Jiangxi Provincial Health Commission (No. 2018b068) and Science and Technology Plan of Jiangxi Provincial Health Commission (No. 20203077).

## Conflict of Interest

The authors declare that the research was conducted in the absence of any commercial or financial relationships that could be construed as a potential conflict of interest.

## Publisher’s Note

All claims expressed in this article are solely those of the authors and do not necessarily represent those of their affiliated organizations, or those of the publisher, the editors and the reviewers. Any product that may be evaluated in this article, or claim that may be made by its manufacturer, is not guaranteed or endorsed by the publisher.
